# High rate of marginal vitamin A deficiency in children aged 0–6 years in Quanzhou, China: a cross-sectional study

**DOI:** 10.3389/fpubh.2026.1727761

**Published:** 2026-03-25

**Authors:** Xiaoyan Zhou, Xiaoyan Zhuang, Huiling Huang, Jiajia Liu, Qingling Zhu

**Affiliations:** 1 Department of Children Health Care, Quanzhou Women and Children's Hospital, Quanzhou, Fujian, China; 2 The Graduate School of Fujian Medical University, Fuzhou, Fujian, China; 3 The School of Clinical Medicine, Fujian Medical University, Fuzhou, Fujian, China; 4 Department of Women’s Health Section, Quanzhou Women and Children’s Hospital, Quanzhou, Fujian, China

**Keywords:** children, nutritional status, serum retinol, supplementation, vitamin A

## Abstract

**Objective:**

To evaluate the vitamin A nutritional status and identify associated factors among children aged 0–6 years in Quanzhou, China, to support the development of targeted intervention strategies.

**Method:**

This study included 1,183 healthy children (0–6 years) from January 2022 to March 2023. Serum retinol was measured via LC–MS/MS, and anthropometric data were collected. The vitamin A status was compared by gender, age and BMI.

**Results:**

The median serum retinol concentration was 1.12 μmol/L (95% CI: 0.94–1.29). The overall prevalence of marginal deficiency or deficiency (serum retinol <1.05 μmol/L) was 38.89% (460/1183), with the highest rate observed in infants (65.87%, 166/252), followed by preschoolers (35.74%, 168/470) and toddlers (27.33%, 126/461). Age was a significant predictor of vitamin A sufficiency (*p* < 0.001): toddlers and preschoolers had 5.06 and 3.33 times higher odds of vitamin A sufficiency compared to infants, respectively. Overweight/obese children showed a higher rate of normal vitamin A status than those with normal or underweight BMI (*p* < 0.05). No significant differences were found by sex (*p* > 0.05).

**Conclusion:**

There is a concerning high prevalence of marginal vitamin A deficiency among young children in Quanzhou, representing a notable public health issue, particularly severe in infants. Interventions should be age-specific and incorporate BMI-related metabolic considerations to effectively address vitamin A insufficiency in this and similar populations.

## 1 Introduction

Vitamin A is an essential nutrient for infants and young children, playing critical roles in growth, development, and overall health. As a fat-soluble vitamin, it can be stored in the body for extended periods. It is vital for maintaining healthy vision, supporting immune function, and promoting proper growth and development. Vitamin A occurs in two primary forms in foods: preformed vitamin A (retinol), derived from animal sources, and provitamin A carotenoids from plant-based foods. Rich dietary sources include liver, fish, dairy products, fortified cereals, and orange or yellow fruits and vegetables such as carrots, sweet potatoes, and mangoes (1, 2). Deficiency can lead to visual impairments, compromised immunity, and growth retardation.

Quanzhou, a historic coastal city in Fujian Province, has a traditional dietary pattern rich in seafood, poultry, and locally grown vegetables such as sweet potatoes, carrots, and leafy greens—all potential sources of provitamin A carotenoids. However, rapid urbanization and economic growth have shifted diets toward processed and convenience foods, which are often energy-dense but micronutrient-poor. This transition, coupled with inadequate dietary diversity and low awareness of nutrient-rich foods, is a recognized risk factor for micronutrient deficiencies such as vitamin A deficiency (VAD) (3). This rising prevalence of childhood obesity alongside persistent micronutrient deficiencies illustrates a dual burden that complicates public health responses. Importantly, VAD in children is associated with increased mortality from infectious diseases (4). Compared to megacities such as Beijing and Shanghai, where comprehensive fortification programs and nutrition surveillance are well-established, Quanzhou—like many third-tier cities in China—faces fragmented policy implementation and limited resources for child nutrition interventions. These regional specificities udnerline the need for context-sensitive strategies, such as community-based supplementation, tailored nutrition education, and improved food fortification. Evidence suggests that community-level nutrition education is crucial for improving dietary practices (3), and a shift from universal to targeted supplementation may be beneficial in certain settings (4). The effectiveness of interventions also depends on the baseline risk of the target population, highlighting the importance of well-designed, context-specific strategies (5).

Globally, an estimated 30% of children under five are deficient in vitamin A, accounting for about 2% of deaths in this age group (6). Vitamin A status has been linked to various health conditions, including autism spectrum disorders (7), respiratory infections (8, 9), asthma (10) and malaria (11). In 2013, the WHO classified VAD as a public health issue affecting about one-third of children aged 6–59 months, with the highest prevalence in sub-Saharan Africa (48%) and South Asia (44%) (6). For instance, a study in Iran reported a VAD rate of 18.3% among children aged 15–23 months in 2012, a sharp rise from 2.1% in 2001 (12). The global prevalence of VAD (serum retinol <0.7 μmol/L) among children under five was 29% in 2013 (6, 13). Periodic high-dose vitamin A supplementation is a proven, low-cost intervention that reduces all-cause mortality by 12–24% and remains a critical component of global child survival efforts (13).

Vitamin A requirements vary across geographic and socioeconomic contexts. In high-income countries such as the United States and members of the European Union, widespread food fortification and diverse diets keeps marginal VAD rates below 5% (14). In contrast, many low- and middle-income countries, particularly in sub-Saharan Africa and South Asia, continue to face a high burden of VAD, with prevalence rates exceeding 40% (6). Similar disparities exist within China. Studies from first-tier cities like Beijing and Shanghai report lower prevalence of vitamin A insufficiency, likely due to better economic conditions and healthcare access, whereas research from less developed regions such as rural Western China indicates higher deficiency rates (15).

Quanzhou, a mid-sized coastal city undergoing rapid urbanization, represents an understudied setting that bridges affluent metropolitan areas and resource-limited rural regions. Its nutritional profile—characterized by coexisting micronutrient deficiencies and rising childhood obesity (16, 17)—provides a unique context in which to examine vitamin A status within an emerging economy. While national epidemiological surveys have broadly characterized VAD among Chinese children, data from rapidly urbanizing mid-sized cities remain sparse. A systematic review and modeling analysis published in 2023, covering low - and middle-income countries (LMICs), including China, confirmed that the burden of VAD is higher in areas with lower Socio-Demographic index (SDI) (corresponding to underdeveloped areas), with a prevalence rate of 29.67% in low-SDI areas and only 5.17% in high-middle SDI areas. Meanwhile, the prevalence of VAD is highest in the younger age group (such as children aged 0–5), reaching 19.53%, highlighting an age-related difference of 2 years. In addition, research has pointed out that the lack of reliable data globally hinders the progress of VAD prevention and control, and the representativeness of data in many regions (including coastal cities undergoing economic transformation) is insufficient (18).

This study aims to address this gap by providing the first comprehensive, age- and BMI-stratified analysis of serum retinol levels in children aged 0–6 years in Quanzhou. By identifying subgroup-specific risk profiles—particularly the high burden of marginal VAD in infants and its paradoxical association with overweight—this work seeks to inform targeted, context-sensitive interventions for similar transitional urban populations in China and beyond.

## 2 Methods

### 2.1 Participants

This cross-sectional study was based on primary data collection from children aged 0–6 years who underwent routine physical examinations at the Child Health Department of Quanzhou Women and Children’s Hospital between January 2022 and March 2023. Vitamin A levels were obtained through direct blood sample testing, and anthropometric data (height and weight) were measured during the clinical visit. No medical record data were used for these primary outcomes. Inclusion criteria were: (1) healthy children aged 0–6 years; (2) availability of serum vitamin A test results; and (3) signed informed consent from guardians. Exclusion criteria were: (1) diagnosis of VAD or borderline deficiency within the past 6 months accompanied by high-dose vitamin A supplementation; and (2) presence of infectious diseases, organ dysfunction, congenital disorders, or inherited metabolic diseases. The study was approved by the Ethics Committee of Quanzhou Women and Children’s Hospital (Approval No. 202013), and informed consent was obtained from all guardians.

The sample size was calculated based on an expected prevalence of VAD of 10% among Chinese children (19), with a 95% confidence level and a 5% margin of error. The initial estimate yielded 139 participants. To account for stratified sampling (design effect = 1.5) and a 20% dropout rate, the minimum sample size was adjusted to 261 per age stratum (infants, toddlers, preschoolers). During the study period, consecutive enrollment resulted in 252 infants, 461 toddlers, and 470 preschoolers, totaling 1,183 participants—exceeding the required sample size and providing a predetermined difference in detection ability of >99%.

### 2.2 Vitamin A detection and sample grading

Vitamin A levels were assessed through an enzyme-linked immunosorbent assay of serum samples. Fasting venous blood samples (3 mL) were collected from each participant in the early morning. Samples were centrifuged at 3500 rpm for 15 min, and serum was aliquoted for analysis. Serum vitamin A (retinol) concentration was measured using enzyme-linked immunosorbent assay (ELISA) kits manufactured by Hefei Harmony Medical Technology Co., Ltd. (Anhui, China). All testing was conducted by Hehe Medical Laboratory (Anhui, China). Both inter- and intra-assay coefficients of variation were below 10%. All testing was conducted by Hangzhou Hehe Medical Laboratory. Vitamin A status was classified based on serum retinol levels.

### 2.3 Standardized anthropometric data collection

All anthropometric data were collected prospectively during the clinical visit by trained staff, not retrieved from medical records. Recumbent length was measured to the nearest 0.1 cm using an infantometer (model WS-RTG-1, Kangwa, China) for children under 2 years of age. Standing height was measured to the nearest 0.1 cm using a stadiometer (model RTCS-090-A, Telecommunications Factory 7, China) for children aged 2 years and older. Weight was measured to the nearest 0.1 kg using a calibrated electronic scale (model RTCS-090-A, Telecommunications Factory 7, China), with participants wearing light clothing.

### 2.4 Group assignment methodology

Participants were stratified into three age groups: infants (≤1 year), toddlers (>1–3 years), and preschoolers (>3–6 years) based on key developmental and dietary transition stages (20) relevant to vitamin A metabolism and intake patterns. Serum retinol levels were interpreted using established thresholds: deficiency (<0.70 μmol/L), marginal deficiency (0.70–1.05 μmol/L), and normal (≥1.05 μmol/L) (21–23).

Nutritional status was assessed according to the 2022 Expert Consensus on the Diagnosis, Evaluation and Management of Childhood Obesity in China (23).

Body mass index (BMI; kg/m^2^) was used for children aged 2 years and older. For children aged 2–5 years, BMI cut-offs from the Growth Curves of Body Mass Index for Chinese Children and Adolescents Aged 0–18 Years were applied (24). For children who had reached 6 years of age, gender- and age-specific BMI references from the Screening for Overweight and Obesity in School-Aged Children and Adolescents were applied (25). For children under 2 years, weight-for-length Z-scores (WLZ), based on the WHO 2006 Child Growth Standards, were employed. Nutritional status categories were defined as: overweight/obese (WLZ > +2 SD), underweight (WLZ < −2 SD), and normal (−2 SD ≤ WLZ ≤ +2 SD) (26) to align with clinically recognized cut-offs for underweight, normal weight, and overweight/obesity.

### 2.5 Statistical analysis

Data analysis was performed using SPSS software (version 26.0). Continuous variables with normal distribution were presented as mean ± standard deviation (SD), and comparisons between two groups were conducted using independent samples t-test, while one-way ANOVA or ANCOVA was applied for comparisons among multiple groups. Normality was assessed using the Shapiro–Wilk test. Non-normally distributed continuous variables were expressed as (*P_25_ ~ P_75_*), with group differences assessed using nonparametric tests: the Mann–Whitney U test for two-group comparisons and the Kruskal–Wallis H test for multiple groups. Categorical variables were summarized as frequencies and percentages. For comparisons of categorical variable, Pearson’s chi-square test was used when the expected count in all cells was ≥5. For subgroups with small sample sizes where the expected count was <5, Fisher’s exact test was employed to ensure statistical validity.

Linear regression was used to examine the effects of explanatory variables on continuous outcomes. Multivariable binary logistic regression was employed to identify independent factors associated with vitamin A sufficiency (defined as serum retinol ≥1.05 μmol/L), with adjustment for age group, sex, and BMI category. Results are reported as adjusted odds ratios (*aOR*) with 95% confidence intervals (*CI*). Age group was incorporated as an ordinal variable (infant → toddler → preschooler) to reflect developmental trends. BMI and WLZ were divided into two categories based on age- and sex- specific Z-scores: overweight/obese (BMI > +2 SD or WLZ > +2) and normal/underweight (BMI ≤ +2 SD, or WLZ ≤ +2). The use of binary BMI/WLZ categories in regression models aimed to maximize statistical power and simplify the interpretation of the association with vitamin A sufficiency. A two-sided *p*-value < 0.05 was considered statistically significant.

## 3 Results

### 3.1 General information

The demographic and baseline characteristics of the 1,183 participants are summarized in Table 1. The sample comprised 704 boys and 479 girls, with a median age of 2.00 (1.0–4.0) years. Participants were stratified into three age groups: infants (0–1 year; *n* = 252), toddlers (1–3 years; *n* = 461), and preschoolers (3–6 years; *n* = 470). Sex distribution did not differ significantly across age groups (*χ*^2^ = 58.552, *p* > 0.05). Anthropometric categories are also presented in Table 1.

**Table 1:** Serum vitamin A levels.

Grouping		N	Vitamin A (μmol/L)	*Z*	*p*	Vitamin A nutritional status (%)			*χ* ^2^	*p*
						Deficiency	Marginal deficiency	Normal		
All the children		1,183	1.12 (0.94–1.29)			48 (4.06)	412 (34.83)	723 (61.11)	578.90	<0.001
Sexes	Male	704	1.12 (0.94 ~ 1.29)	160887.000	0.180	31 (4.40)	252 (35.80)	421 (59.80)	1.473	0.479
	Female	479	1.12 (0.98 ~ 1.33)			17 (3.55)	160 (33.40)	302 (63.05)		
Seasons	Spring	85	1.12 (0.93 ~ 1.31)	5.329	0.149					
	Summer	297	1.15 (0.98 ~ 1.33)							
	Autumn	405	1.15 (0.98 ~ 1.29)							
	Winter	396	1.12 (0.91 ~ 1.29)							
Age	Infant	252	0.94 (0.80 ~ 1.12)	144.836	<0.001	29 (11.50)	137 (54.37)	86 (34.13)	124.017	<0.001
	Toddler	461	1.22 (1.05 ~ 1.36)			7 (1.52)	119 (25.81)	335 (72.67)		
	Preschooler	470	1.15 (0.98 ~ 1.33)			12 (2.55)	156 (33.19)	302 (64.26)		
Weight-for-length Z csore	<−2	19	1.08 (0.87 ~ 1.43)	0.009	0.995	2 (10.53)	7 (36.84)	10 (52.63)	4.888	0.243
	≥ − 2, ≤2	531	1.12 (0.91 ~ 1.29)			28 (5.27)	209 (39.40)	294 (55.36)		
	>2	10	1.10 (0.74 ~ 1.57)			2 (20)	3 (30)	5 (50)		
BMI	Underweight	29	1.12 (0.96 ~ 1.38)	4.436	0.019	0 (0.00)	19 (37.93)	64 (77.11)	6.620	0.129
	Normal weight	511	1.15 (0.98 ~ 1.29)			15 (2.94)	163 (31.90)	333 (65.17)		
	Overweight/obese	83	1.19 (1.08 ~ 1.39)			1 (3.45)	11 (37.93)	17 (58.62)		

Among children under 2 years of age, 19 had a WLZ < −2, 531 had WLZ between −2 and +2, and 10 had WLZ > +2. For children aged ≥2 years, 29 were underweight, 511 had normal weight, and 83 were overweight/obese. The overall vitamin A level was 1.12 (0.91–1.29) μmol/L, with no significant differences between groups (*p* > 0.05). Among children aged ≥2 years, the Vitamin A level was 1.10 (1.01–1.33) μmol/L, also with no significant intergroup differences (*p* > 0.05).

### 3.2 Analysis of vitamin A levels in children aged 0–6 years

**Figure 1:**
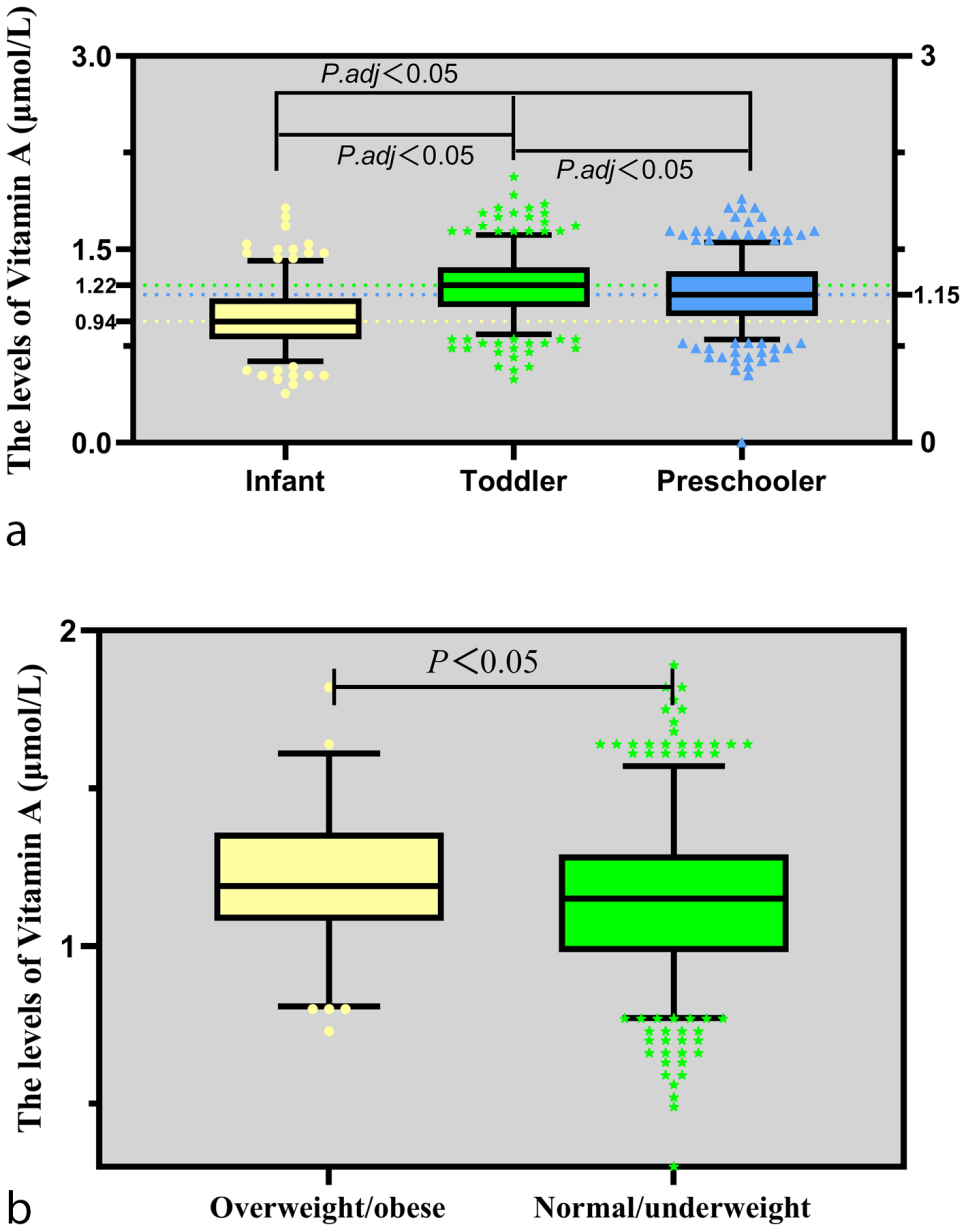
Levels of vitamin A. **(a)** Comparison of vitamin A levels in children of different ages; **(b)** Comparison of vitamin A levels in children with varying BMIs.

The median serum vitamin A concentration for all children was 1.12 (0.94–1.29) μmol/L. No significant sex-based difference was observed: boys, 1.12 (0.94–1.29) μmol/L; girls, 1.12 (0.98–1.33) μmol/L (*Z* = 160887.00, *p* > 0.05). However, vitamin A levels differed significantly by age groups (*Z* = 144.836, *p* < 0.001), with infants showing the lowest levels, followed by preschoolers, and toddlers the highest (Figure 1a). No significant variations were observed by WLZ categories (*Z* = 0.009, *p* > 0.05). Notably, overweight/obese children had significantly higher vitamin A levels compared to normal/underweight status (*Z* = −2.102, *p* = 0.036) (Figure 1b).

### 3.3 Vitamin A status in children aged 0 ~ 6 years

**Figure 2:**
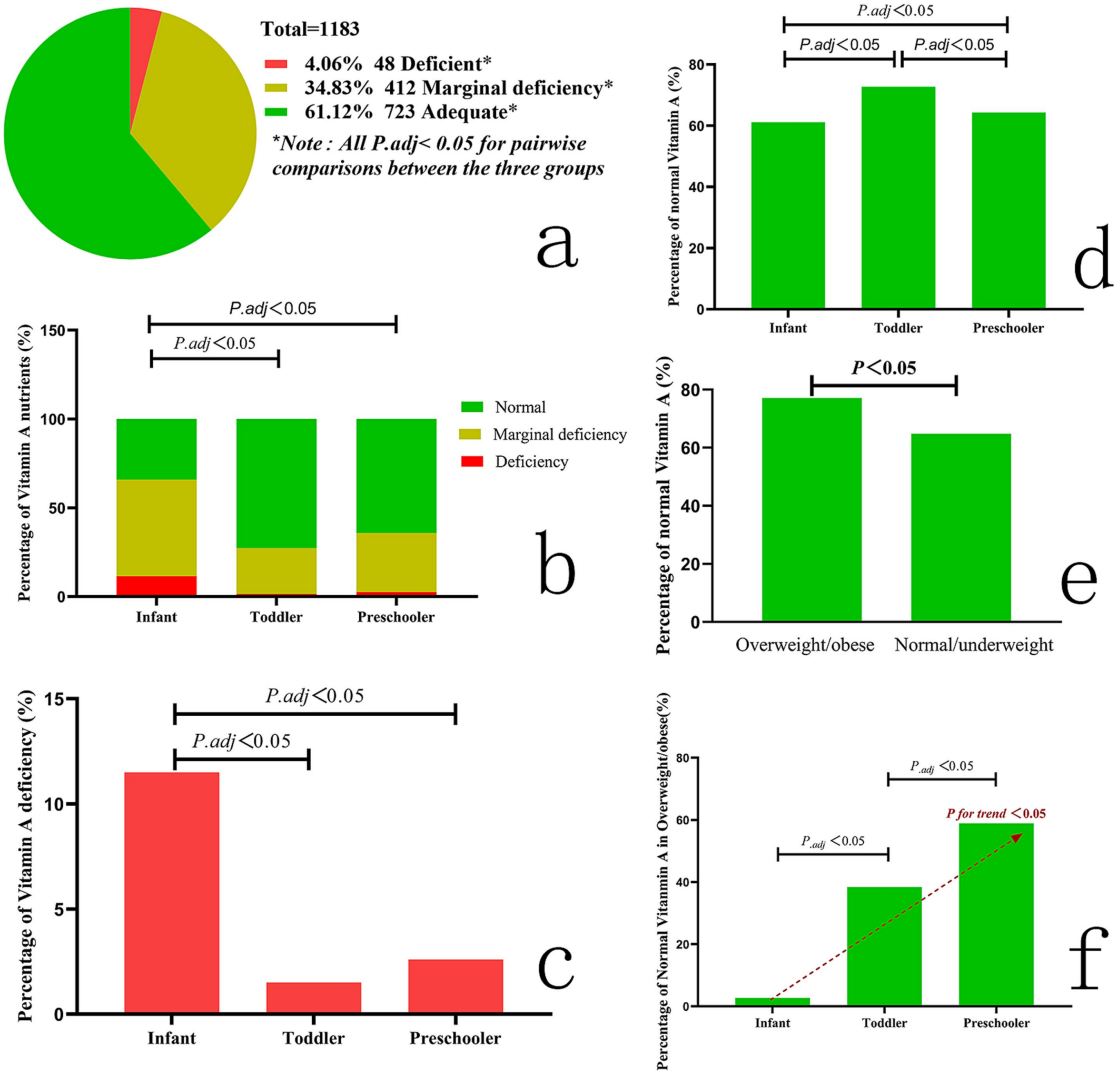
Composition of nutritional status of vitamin A. **(a)** Composition ratio of vitamin A for all children; **(b)** composition of vitamin A nutritional status in children of different ages; **(c)** comparison of vitamin A deficiency rates in children of different ages; **(d)** comparison of normal vitamin A rates in children of different ages; **(e)** comparison of vitamin A normal rates in children with varying BMIs; **(f)** percentage of normal vitamin A in overweight/obese.

The prevalence of VAD, marginal deficiency, and normal status across the total sample and subgroups is detailed in Table 1. The composition ratio differed significantly among these groups (*p* < 0.001; Figure 2a), and all pairwise comparisons were statistically significant (all *P_.adj_* < 0.001).

Significant differences were observed across age groups (*p* < 0.001; Table 1, Figure 2b), with infants showing the highest deficiency rate (11.50%) and toddlers the highest normal rate (72.67%). Additionally, the proportion of children with normal vitamin A status was lowest in infants, intermediate in preschoolers, and highest in toddlers (all *p* < 0.05; Table 1, Figures 2c,d).

Due to limited sample sizes in extreme WLZ and BMI categories (*n* < 5 in some subgroups), *Fisher’s exact test* was employed comparisons as appropriate. However, no differences in vitamin A nutritional status were found among overweight/obese, normal weight, and underweight children (see Table 1). Comparisons of the proportion of children with normal vitamin A status showed no significant differences by WLZ category (*χ*^2^ = 1.240, *p* > 0.05) or BMI category (*χ*^2^ = 4.304, *p* > 0.05). Among children <2 years, no difference was found between those with WLZ > 2 and WLZ ≤ 2 (*χ*^2^ = 0.110, *p* > 0.05). In contrast, among children ≥2 years, overweight/obese individuals had a significantly higher proportion of normal vitamin A status than those with normal or underweight BMI (*χ*^2^ = 4.877, *p* < 0.05; Figure 2e). Moreover, the proportions of normal vitamin A status among overweight/obese children increased with age (*χ*^2^ = 9.265, *p* < 0.05; Figure 2f).

### 3.4 Factors associated with normal vitamin A levels

**Figure 3:**
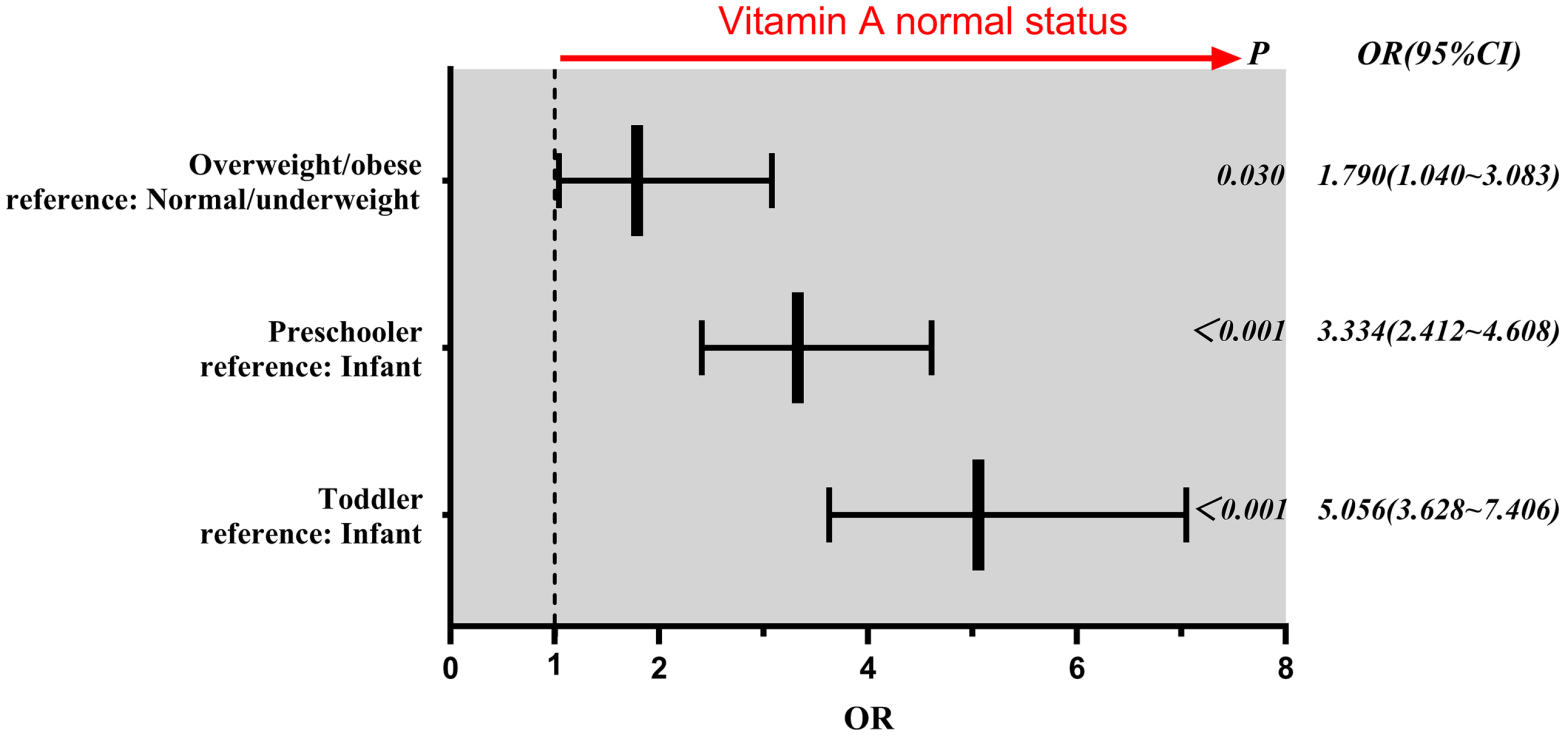
Forest map of factors influencing vitamin A normal status.

Binary logistic regression identified age and BMI as independent predictors of vitamin A sufficiency (Figure 3). Compared to infants, toddlers (*aOR* = 5.06; 95% CI: 3.63–7.05; *p* < 0.001) and preschoolers (*aOR* = 3.33; 95% CI: 2.41–4.61; *p* < 0.001) had significantly higher odds of normal vitamin A status. Overweight/obese children had 1.8-fold higher odds of sufficiency than normal/underweight children (*aOR* = 1.79; 95% CI: 1.04–3.08; *p* = 0.03), after adjusting for age and sex.

## 4 Discussion

This study focuses on the critical developmental window of ages 0 to 6 years, differing from previous nutritional surveys in Quanzhou, which primarily targeted school-aged children. The developmental window from birth to 6 years is characterized by rapid growth and heightened susceptibility to micronutrient deficiencies. Our findings reveal a unique nutritional paradox—defined as the simultaneous presence of contrasting nutritional problems—in this transitioning urban environment, where a high prevalence of marginal VAD coexists with rising rates of childhood obesity. This paradox highlights the need for dual-targeted public health strategies that address both micronutrient deficiencies and obesity concurrently.

This retrospective analysis assessed the vitamin A nutirtional status of children aged 0–6 years in Quanzhou, a third-tier city in China, over a one-year period. The median vitamin A level was 1.12 (0.94–1.29) μmol/L, exceeding the normal threshold of 0.70 μmol/L. The results indicated that vitamin A levels in Quanzhou fell between those by Chen et al. (15) for a fifth-tier Chinese city (1.16 μmol/L) and a low-income country (1.03 μmol/L). The prevalence of VAD and marginal deficiency in this study (4.06 and 34.83%, respectively) were slightly higher than those reported by Chen et al. (15) (4.06% *vs.* 2.64; 34.83% *vs.* 32.86%, respectively). In contrast, the prevalence of VAD in our study (4.06%) was significantly lower than the 29.0% reported in 2015 for children aged 6–59 months in low- and middle-income countries (6).

The marginal VAD prevalence found in this study was 38.89%, aligning with the national estimate of approximately 35% reported by Song et al. (19). However, the prevalence of deficiency in infants (65.87%) exceeded rates observed in more economically developed regions such as Beijing and Shanghai, highlighting the pronounced vulnerability of this group in transitional urban settings. Compared to data from western rural China (15), Quanzhou exhibits a distinct profile: lower rates of clinical VAD but higher rates of marginal deficiency. This pattern may reflect an ongoing dietary transition toward energy-dense but micronutrient-poor foods. Furthermore, although not systematically analyzed in this study, preliminary data suggest a co-occurrence of vitamin A and vitamin D insufficiency in a subset of participants, consistent with the 2024 review by Palmer et al. on the synergistic roles of these nutrients in child health (27). Future studies should explicitly explore this interplay in similar populations.

Our findings partially align with those of Chen et al. (15), who also identified age as a significant predictor of vitamin A status in central and western China. However, Chen et al. (15) observed a gradual increase in serum vitamin A levels with age. They also noted a concurrent rise in vitamin A insufficiency among older children—a trend not observed in our cohort. This discrepancy may reflect regional differences in dietary patterns, socioeconomic status, or healthcare access. Specifically, these differences exist between Quanzhou, a coastal city classified as third-tier in China’s urban hierarchy, and the less developed regions studied by Chen et al. notably, our study identified infants as the most vulnerable subgroup. They exhibited a marginal VAD rate of 65.9%, a figure substantially higher than the 39.0% reported in a supplementation trial among 6-month-old infants in Chongqing (28). This suggests that without systematic perinatal or early infancy supplementation, infants in rapidly developing urban areas like Quanzhou remain at high risk.

Placing Quanzhou within the context of recent studies on transitional Chinese cities further elucidates its unique nutritional profile. A large multicenter survey conducted across 20 cities in China, published in 2023, reported an overall vitamin A insufficiency rate of 29.27% among preschool children aged 2 to 6 years, with higher rates observed in older children and those from lower-income families (29). Another study from Hainan Province in 2024 focusing on children aged 0–3 years found a marginal VAD rate of 24.73%, with infants aged 0–6 months showing the highest burden (45.98% marginal deficiency, and 22.99% deficiency) (30). This aligns with earlier findings from central and western China, where children in less developed regions and younger age groups exhibited significantly higher rates of vitamin A insufficiency (15). Our findings from Quanzhou, a coastal third-tier city, reveal a marginal VAD rate of 38.89%, which is notably higher than the national multicenter average. This further underscores the pronounced vulnerability of infants, with 65.87% exhibiting marginal or deficient status. These comparisons highlight that rapidly urbanizing settings like Quanzhou may experience an accentuated burden of marginal VAD, particularly during early infancy. This underscores the need for targeted, age-stratified interventions in similar transitional urban contexts. Furthermore, our data on the association between overweight/obesity and higher vitamin A sufficiency provide additional insight into the “double burden of malnutrition”—the coexistence of undernutrition and over nutrition—in these settings. This phenomenon is increasingly reported in transitional economies but has been less frequently examined at the micronutrient level in early childhood (31).

Internationally, similar age-related patterns of VAD have been documented. In Bangladesh, a complementary food supplementation trial among 18-month-old children demonstrated that intervention groups achieved significantly higher serum retinol levels (1.23–1.28 μmol/L) than controls (1.13 μmol/L), underscoring the effectiveness of structured nutritional support during the weaning period (32). In contrast, in sub-Saharan Africa—where VAD remains a severe public health burden—studies such as those from Nigeria report deficiency rates as high as 68.2% among infants. This stark difference highlights how regional disparities in infrastructure, food security, and supplementation coverage can exacerbate nutritional vulnerability (33).

Age was identified as a significant factor influencing vitamin A levels. Our results showed that toddlers had the highest vitamin A levels, followed by preschoolers, while infants had the lowest. The prevalence of VAD was lowest in infants (11.5%), increased to 27.3% in toddlers and 35.8% in preschoolers. In the study by Chen et al. (15), serum vitamin A levels increased with age, but the risk of VAD also rose with age—a trend not observed in our cohort. The high rate of VAD among infants in the present study, counting for more than half of all insufficiency cases, underscores this group’s vulnerability, although rates declined notably in toddlers and preschoolers. The significantly higher vitamin A levels observed in 6-month-old infants from Chongqing (1.06 μmol/L) (28) compared to the 0–1 year cohort in Quanzhou (0.94 μmol/L) may be attributed to early supplementation strategies. In the Chongqing study, some newborns received weekly vitamin A supplements exceeding 1,500 IU shortly after birth, with supplementation rates gradually increasing during follow-up. This proactive approach reduced the marginal deficiency rate to 39.0% by 6 months of age—markedly lower than the 65.9% rate observed in Quanzhou. This disparity highlights the potential efficacy of perinatal vitamin A supplementation in mitigating deficiency during the critical weaning period, a strategy not currently integrated into routine care in Quanzhou. Similarly, a complementary food supplementation trial among 18-month-old rural Bangladeshi children demonstrated significantly higher inflammation-adjusted serum retinol levels in intervention groups (1.23–1.28 μmol/L) compared to controls (1.13 μmol/L), resulting in a low post-intervention VAD prevalence of 7.9% (32). These comparisons emphasize the severity of vitamin A insufficiency among infants in Quanzhou and support the potential effectiveness of structured, fortified complementary feeding programs similar to those implemented in Bangladesh. The rate of vitamin A insufficiency in this study was lower than that reported by Chen et al. (15) for children of similar ages (28.92% at 3 years, 38.38% at 4 years, and 42.37% at 5–6 years). Globally, VAD is most prevalent among children under 5 years of age, affecting an estimated 190 million individuals, primarily in Africa and Southeast Asia (33). The age-related trends observed in our study highlight the dynamic nature of nutritional requirements during childhood and underscore the importance of age-specific interventions to optimize vitamin A status.

The steep gradient in deficiency rates between infants (11.5%) and toddlers (1.52%) likely reflects transitional challenges during weaning, including diets low in preformed vitamin A and immaturity of hepatic vitamin A storage. The combined rate of marginal and clinical VAD among infants in Quanzhou reaches 65.9%, representing an urgent public health issue. To address this issue, the following measures are recommended for Quanzhou and comparable third-tier cities: (a) integrate vitamin A status screening into routine infant health check-ups, particularly during the critical window of 6–12 months of age; (b) enhance nutritional education for caregivers, stressing the timely introduction of complementary foods rich in vitamin A and beta-carotene (e.g., animal liver, deeply colored vegetable and fruit purées); and (c) evaluate the feasibility and necessity of high-dose vitamin A supplementation programs for infants, taking into account WHO recommendations for high-burden regions and adapting them to local conditions.

In this study, overweight/obese children exhibited significantly higher vitamin A levels than those with normal or underweight BMI. This association may be influenced by factors such as dietary intake, metabolic variations, and adipose tissue storage capacity, given the fat-soluble nature of vitamin A. However, emerging evidence indicates that retinol sequestration in adipose tissue may lead to functional VAD—characterized by impaired vitamin A activity despite apparently adequate circulating levels (34, 35). This paradox highlights the need for BMI-stratified reference ranges in populations experiencing rapid nutritional transition. Considering the higher prevalence of vitamin A sufficiency alongside the potential risk of functional deficiency among overweight/obese children, the following actions are recommended: (a) public health monitoring systems should incorporate both vitamin A status and obesity indicators to enable a more comprehensive nutritional assessment; (b) nutrition intervention strategies should be inclusive and ensure coverage of children across all weight categories, with particular attention to micronutrient status in overweight and obese individuals; and (c) research should be intensified to identify better biomarkers or methods for assessing functional vitamin A status in children with obesity.

The marginal VAD rate of 38.89% observed in this study exceeds the national rate of 31.53% reported in the 2024 Chinese expert consensus (36). This higher prevalence likely reflects the rapid dietary transition in third-tier cities, which are typically defined by their population size and economic development level, such as Quanzhou, where shifts toward processed foods may outpace nutritional awareness and contribute to ongoing deficiencies despite economic growth. While the consensus notes an age-related increase in marginal deficiency, our study found the highest burden in infancy without a consistent rise in older age groups. This difference may be related to local feeding practices or healthcare focus and warrants further investigation. Overall, our data demonstrate that the national trend of marginal VAD is present and even accentuated in rapidly urbanizing third-tier settings.

Placing our findings within the context of local nutritional research in Quanzhou provides deeper insights. The 2022 Nutritional Status Analysis of Primary School Students in Licheng District, Quanzhou reported a dual burden of malnutrition (11.62% in suburban groups), coexisting with overweight/obesity (20.64% overweight, and 17.64% obesity in urban groups) among school-aged children (37). Our study reveals a corresponding nutritional paradox in children aged 0–6 years. There is a high prevalence of marginal VAD, particularly severe among infants (65.87%), while overweight/obese children show a higher rate of vitamin A sufficiency compared to their peers. Together, these two studies outline a persistent dual nutritional challenge for children in Quanzhou from early childhood to school age: the co-existence of micronutrient deficiencies and over nutrition. This highlights the need for twin-track public health strategies that simultaneously address the prevention of micronutrient deficiencies (focusing on infants and children in under-resourced areas) and the management of overweight/obesity in all children, while remaining vigilant about potential hidden hunger (micronutrient inadequacy) among those who are obese.

As fat-soluble vitamins, vitamin A and the vitamin D exhibit significant synergistic roles in immune regulation, bone development, and cellular growth. The 2024 expert consensus explicitly states that the presence of vitamin A can enhance the activity of the vitamin D receptor (VDR), increasing the biological effects mediated by VDR by up to 130%. Furthermore, combined supplementation of vitamins A and D may more effectively improve children’s nutritional status (36). Substantial literature documents that deficiencies of vitamin A and the vitamin D frequently co-occur, particularly among children with infectious diseases, such as recurrent respiratory tract infections, and neurodevelopmental disorders, including autism spectrum disorder (7, 27).

Our study found an exceptionally high burden of VAD in infancy. Given that vitamin D deficiency is also highly prevalent among Chinese children (36), and considering their shared risk factors (e.g., insufficient maternal stores transferred prenatally, rapid growth during infancy, and inadequate dietary intake), it is reasonable to speculate that a significant proportion of our study population may have concurrent deficiencies of both vitamin A and D. Although vitamin D levels were not systematically assessed in this study, this potential dual deficiency should not be overlooked. Future research should concurrently evaluate the status of both vitamin A and D to provide a more comprehensive picture of children’s micronutrient nutrition. Based on these findings, we suggest that in Quanzhou and similar settings, exploring a combined vitamin A and D supplementation strategy as part of routine child healthcare for high-risk infant groups may yield superior synergistic health benefits compared to single-nutrient supplementation. This approach would also be consistent with principles of cost-effectiveness.

No significant differences in Vitamin A levels were observed between males and females in this study. The Estimated Average Requirements (EARs) for vitamin A intake established by the Office of Dietary Supplements indicate no sex-based differences for children up to 8 years of age. These EARs are 400 mcg for 0–6 months, 500 mcg for 7–12 months, 300 mcg for 1–3 years, and 400 mcg for 4–8 years (38). Notably, the EAR for children aged 1–3 years is the lowest among the 0–6 age group. Our findings, which show the highest vitamin A levels in children aged 1–3 years and no significant sex-based differences, align with these recommendations and existing knowledge of vitamin A physiology in children. The observed age-related variations reflect differential vitamin A requirements across developmental stages, supporting the physiological basis for our findings.

The elevated vitamin A levels observed in overweight/obese children are consistent with previous studies. For instance, children with obesity—particularly those with abdominal obesity—have been shown to exhibit higher serum vitamin A levels (34). Several studies have reported significant associations between elevated vitamin A levels and an increased risk of childhood obesity (39, 40). Furthermore, a study involving children and adolescents from Eastern China revealed positive correlations between vitamin A levels and obesity, metabolic syndrome, dyslipidemia, and hyperuricemia (41). The intriguing finding of higher vitamin A levels in overweight/obese children aligns with growing evidence of a complex interplay between vitamin A metabolism and adiposity (2, 35, 39). Further investigation is required to elucidate the underlying mechanisms. Nonetheless, our study contributes to the evolving understanding of how weight status influences micronutrient status, particularly vitamin A. Furthermore, our analysis revealed an age-dependent pattern within this association: the rate of vitamin A sufficiency among overweight/obese children increases significantly with age. This finding suggests that the metabolic or dietary factors linking adiposity and vitamin A status may evolve during early childhood. Specifically, for younger overweight/obese children (e.g., infants and toddlers), vitamin A sufficiency is lower, potentially reflecting poorer dietary diversity or stronger sequestration of retinol in developing adipose tissue. In older preschool-aged children, higher sufficiency rates may correspond to greater consumption of vitamin A-rich foods, better hepatic storage capacity, or differences in fat metabolism related to vitamin A mobilization. This age–BMI interaction underscores the complexity of nutritional status assessment in growing children and highlights the need for age-stratified approaches when evaluating micronutrient status in relation to body weight.

The generally elevated vitamin A levels in our study population suggest an overall adequate vitamin A status in Quanzhou, providing valuable guidance for health policymakers in resource allocation. However, the marked variations by age and BMI call for nuanced strategies. Although the overall deficiency rate (4.06%) is lower than the average for low- and middle-income countries (29.0%) (6), the deficiency rate among infants (11.5%) approaches emergency levels seen in sub-Saharan Africa (e.g., 68.2% among Nigerian infants) (33). This demonstrates how regional averages can mask severe subgroup vulnerabilities in transitioning economies. Moreover, the higher vitamin A sufficiency among overweight/obese children highlights the complex relationship between obesity and micronutrient status in rapidly developing regions. This divergence necessitates dual-focused interventions. First, immediate targeted supplementation for infants under 1 year, where deficiency constitutes an urgent public health threat. Second, integrated surveillance models for secondary cities—defined as mid-sized urban centers experiencing shifts in dietary patterns and lifestyle—undergoing nutritional transition, combining micronutrient screening with obesity prevention programs. Finally, tailored strategies addressing both extremes—infant deficiency and weight-related metabolic disparities—are needed to optimize vitamin A status across preschool children in Quanzhou and similar settings globally.

Despite its contributions, this study has several limitations that warrant acknowledgment. First, its retrospective design precludes causal inferences between the identified factors (e.g., age, BMI) and the vitamin A status outcomes. Moreover, relying on routine health records limited the collection of key social and behavioral determinants. These include household socioeconomic status (such as parental education and income), detailed child feeding practices (including breastfeeding duration, dietary intake patterns, and complementary food composition), and supplementation history, all of which are essential for understanding the structural and behavioral drivers of vitamin A insufficiency. Second, although BMI was associated with vitamin A status, we did not assess dietary intake patterns or adiposity-related metabolic markers, thereby limiting insight into the biological mechanisms underlying the observed obesity–vitamin A paradox—a phenomenon characterized by the coexistence of obesity and VAD. Third, the study was conducted in a single urban center, which may restrict the generalizability of the findings to rural or other regions with distinct socioeconomic and dietary profiles. Fourth, for certain subgroup analyses (e.g., extreme WLZ categories), small sample sizes limited statistical power and precision. Although Fisher’s exact test was applied where appropriate, these results should be interpreted cautiously and validated in larger studies. Finally, despite adjusting for key confounders, unmeasured variables—such as specific dietary habits and breastfeeding duration—may still influence vitamin A status. Future studies should incorporate tools such as food frequency questionnaires and assessments of hepatic retinol stores (e.g., via retinol isotope dilution) to address these gaps. Nevertheless, these limitations do not undermine the primary findings: (1) the age-specific gradient (highest deficiency in infants) was robustly demonstrated and is consistent with physiological mechanisms. (2) The BMI-related paradox (higher sufficiency in overweight/obese children) was statistically significant and supported by existing literature.

Building on these findings and addressing the current gaps, we propose the following specific directions for future research: (1) Longitudinal Studies: Cohort studies tracking children from infancy through early childhood are needed to establish temporal relationships and understand the dynamics of vitamin A status change over time, particularly during critical transition periods like weaning. (2) Intervention Studies: Well-designed trials are warranted to test the effectiveness of context-specific strategies in settings like Quanzhou. These could include evaluating the impact of integrated nutrition education programs for caregivers, the efficacy of targeted (*vs.* universal) vitamin A supplementation for high-risk infants, or the benefits of combined vitamin A and D supplementation. (3) Mechanistic and Comprehensive Investigations: Future studies should combine biomarker assessment with detailed household surveys to incorporate socioeconomic, dietary, and behavioral data. Research should also explore the underlying metabolic mechanisms linking adiposity and vitamin A status, potentially including assessments of dietary intake, adipokines, and functional vitamin A biomarkers. (4) Expanded Geographical Scope: Multi-center studies involving similar transitional cities across China would enhance the generalizability of findings and help identify broader patterns and determinants of micronutrient status in rapidly urbanizing contexts.

In summary, this study addresses a specific gap in regional surveillance by providing detailed data from Quanzhou, a previously understudied third-tier coastal city in transitional urban China. It confirms the persistence of high rates of marginal VAD despite economic growth. The study identifies infancy as a critical window of vulnerability to VAD and highlights the complex interplay between vitamin A status and childhood obesity, which is a hallmark of the nutrition transition.

Based on the key findings, we propose three targeted, locally informed interventions: First, integrate vitamin A screening into routine infant health check-ups (6–12 months), coupled with counseling on complementary feeding using locally available, vitamin A-rich foods such as sweet potatoes, carrots, and egg yolks. Second, pilot community-based nutrition education programs through maternal and child health centers; these programs should focus on balanced weaning and address the dual risks of micronutrient deficiency and obesity. Third, explore the feasibility of a combined vitamin A and D supplementation strategy for high-risk infants, adapting national guidelines to Quanzhou’s dietary and health system context. These evidence-based measures may serve as a practical model for other midsized cities undergoing similar nutritional transitions.

## Data Availability

The datasets presented in this article are not readily available because the datasets used and/or analyzed during the current study are available from the corresponding author on reasonable request. Requests to access the datasets should be directed to QZ, zhu_qingling2012@163.com.
